# Supplementary material for: Exploration of clinicians’ decision-making regarding transfer of patient care from the emergency department to a medical assessment unit: A qualitative study
Source: PLoS One. 2022 Feb 3;17(2):e0263235. doi: 10.1371/journal.pone.0263235 (PMC8812931; doi:10.1371/journal.pone.0263235)
Supplement: S1 File — (DOCX) [file pone.0263235.s001.docx]

**S1. File Focus Group Guide**

(Note: focus groups to be recorded for transcription)

| **Date:** | **Location:** | **Number of participants:** |
| --- | --- | --- |
| **Time commenced:** | **Participant Group:** |  |
| **Time finished:** | **Participant ID Codes:** | |

**________________________________________________________________________________________**

**Prompts for discussion** *(derived from TDF mapped factors affecting admission to Medical Assessment Units)*

1. **What do you see as the role of the [MAU]?**
   1. **What type of patient is appropriate for admission to [MAU]?**
   2. **Is there an admission and discharge policy and what is the outcome of admission to [MAU]?**
2. **Describe the processes for admission and discharge of eligible patients to [MAU].** TDF Domain 1: Knowledge
   1. **How important do you believe a clear line of command in terms of task delegation around the bed allocation process is?**
   2. **Do you believe this clear line of command is evident in the practice area?** TDF Domain 3: Social/professional role and identify (self-standards) & TDF 9: Social influence (norms)
   3. **What are your beliefs around checklists and protocols for use in decision-making for bed allocation?** TDF Domain 3: Social/professional role and identify (self-standards)
3. **How would you describe the role of [MAU] in ensuring quality and safe patient care for acute medical patients?** TDF Domain 5: Beliefs about consequences (anticipated outcomes/attitude) & TDF Domain 6: Motivation and goals (intention)
4. **What impact or involvement do staff with non-clinical backgrounds have on the decision-making processes regarding bed allocation?** TDF Domain 1: Knowledge & Domain 2: Skills & Domain 3: Social/professional role and identify (self-standards) & Domain 4: Beliefs about capabilities (self-efficacy)
   1. **How far do you believe that non-clinical (as well as clinical) skills are required for safe decision-making?** TDF Domain 1: Knowledge & Domain 2: Skills & Domain 9: Social influences (norms)
5. **Do you feel that there is a multidisciplinary approach to care coordination?**
   1. **How do you feel that this approach impacts upon decision-making regarding bed allocation (positive/negative aspects)?** TDF Domain 2: Skills
6. **How important is the setting of goals for individual patients within specified timeframes?**
   1. **How does this aid determination of the suitability of admission to [MAU] or elsewhere?**  TDF Domain 2: Skills
   2. **What impact do you feel that targets have on bed allocation: for example, transferring patients to the first available, rather than the most appropriate, bed?** TDF Domain 5: Beliefs about consequences (anticipated outcomes/attitude)
   3. **What do you feel the impact of the environment (for example, time, pressure, fatigue as well as time of day) has upon decision-making for bed allocation?** TDF Domain 7: Memory, attention and decision processes & TDF Domain 8: Environmental context and resources (environmental constraints) & Domain 10: Emotion & Domain 12: Nature of the behaviours
   4. **What do you think the impact of limited or incomplete medical information has upon decision-making for bed allocation (if any)?** TDF Domain 7: Memory, attention and decision processes
