# Supplementary material for: Exploration of clinicians’ decision-making regarding transfer of patient care from the emergency department to a medical assessment unit: A qualitative study
Source: PLoS One. 2022 Feb 3;17(2):e0263235. doi: 10.1371/journal.pone.0263235 (PMC8812931; doi:10.1371/journal.pone.0263235)
Supplement: Attachment — Submitted filename: Response to Anita Estes-R1.docx [file pone.0263235.s002.docx]

**Response to Anita Estas (email received Wed 21/07/2021 3:54 PM)**

Dear Associate Professor Osborne,

Thank you for submitting your manuscript entitled "Exploration of clinicians’ decision-making regarding transfer of patient care from the emergency department to a medical assessment unit: a qualitative study" to PLOS ONE.  Your manuscript files have been checked in-house but before we can proceed we need you to address the following issues:

| Item No | Comment | Author Response | Changes / Updates |
| --- | --- | --- | --- |
| 1 | 1) Thank you for including your ethics statement on the online submission form:  "The study obtained ethical approval from The Prince Charles Hospital Human Research Ethics Committee (approval number: HREC/16/QPCH/365) and Queensland University of Technology (approval number: 1700000310).  Written consent was obtained from participants prior to the focus groups and confirmed at the start of each focus group." | We had already included a statement about ethical approval in the Methods section, under the subheading Ethics. **See Methods section, subsection Ethics, page 8, lines 147- 149.**  We have added the additional text and sentence requested under subsection “Recruitment and consent for focus groups” as it was the most logical place in the manuscript. | Changes in *italics*:  All eligible participants were provided with a copy of the relevant Participant Information and Consent form(s)  *and informed consent was obtained prior to participation in the focus group.*  *Written consent was obtained from participants prior to the focus groups and confirmed at the start of each focus group prior to participation in the focus group.*    **Location of added text: Section: “Methods”; Subheading: “Recruitment and consent for focus groups”; page 10, lines 184-186.** |
|  | 2) We note your current Data Availability Statement is… Therefore, before we can proceed, please address the following prompts:  2a.) Please upload the minimal data set as Supporting Information files or deposit them to a stable, public repository and provide us with the relevant URLs, DOIs, or accession numbers. For a list of recommended repositories, please see <https://journals.plos.org/plosone/s/recommended-repositories>. | We randomly accessed 10 recently published papers by PloS One and could not see any reference to data availability or supporting files containing minimal data sets.  We have selected option 2b. Please see response below. |  |
|  | b.) If there are ethical or legal restrictions on sharing a de-identified data set, please explain them in detail (e.g., data contain potentially sensitive information, data are owned by a third-party organization, etc.) and who has imposed them (e.g., an ethics committee. Please also provide non-author contact information* for a data access committee, ethics committee, or other institutional body to which data requests may be sent. | We provided an explanation to this query in the Response to Reviewers file and included it in our Revised cover letter.  We subsequently consulted with the research governance office and our HREC (The Prince Charles Hospital Human Research Ethics Committee) about the request from PLoS One and our concerns that sharing data would violate our informed consent agreement with participants. We have received an email from the Research Governance Officer indicating that the consensus view following a departmental meeting resulted in the Governance Office “agree[ing] with our rationale regarding ethical considerations around publishing [our] qualitative data” and has responded, “In this circumstance, it was agreed that you [the authors] should include The Prince Charles HREC committee [TPCH HREC EC00168] as the institutional contact”. Further, we have an email from TPCH HREC “to confirm that TPCH HREC is happy to be nominated as the contact for data requests”. | Data Availability Statement  Requests in writing for data related to the study titled, *Exploration of clinicians’ decision-making regarding transfer of patient care from the emergency department to a medical assessment unit: a qualitative* *study* (HREC approval number HREC/16/QPCH/365) can be sent to:  The Prince Charles Hospital Human Research Ethics Committee (TPCH HREC EC00168)  Attention: Anne Carle, Executive Officer  Research, Ethics and Governance Unit, Building 14  The Prince Charles Hospital  Rode Road  Chermside Qld 4032 Australia  Ph: (07) 3139 4500  [Research.TPCH@health.qld.gov.au](mailto:Research.TPCH@health.qld.gov.au) |
