# Supplementary material for: Exploration of clinicians’ decision-making regarding transfer of patient care from the emergency department to a medical assessment unit: A qualitative study
Source: PLoS One. 2022 Feb 3;17(2):e0263235. doi: 10.1371/journal.pone.0263235 (PMC8812931; doi:10.1371/journal.pone.0263235)
Supplement: Attachment — Submitted filename: Response to Academic Editor and Reviewer_22-12-2021.docx [file pone.0263235.s003.docx]

Response to Academic Editor and Reviewer #3

| **Comments** | **Author Response** | **Change to Manuscript** |
| --- | --- | --- |
| **FROM ACADEMIC EDITOR:** |  |  |
| 1. Most of the comments have been addressed… | We thank the Editor for this acknowledgement. | **No change to manuscript.** |
| 2. […] however, in order for the paper to be published, the Authors are asked to respond appropriately to the comments of Reviewer 3, in particular on sampling size and sampling strategy, using the suggestions given by Reviewer 3. | We believe we have responded appropriately to Reviewer #3’s comments to date and that our responses on the most recent comments (see below) are equally appropriate, particularly in relation to sample size and sampling strategy, using suggestions by Reviewer #3, where deemed appropriate. | Response to comments and changes to manuscript, where applicable, are identified below. |
| 3. The decision is justified on PLOS ONE’s publication criteria. | We believe our manuscript is aligned with the Submission Guidelines in general, but specifically, in relation to the special section called **Qualitative Research** as outlined below:  **Qualitative research**  “*Qualitative research studies use non-quantitative methods to address a defined research question that may not be accessible by quantitative methods, such as people's interpretations, experiences, and perspectives…”*  A point we made and support with evidence from the literature in our previous responses.  *“…The analysis methods are explicit, systematic, and reproducible, but the results do not involve numerical values or use statistics…”*  We believe we have sufficiently described the explicit and systematic approach of our methodology and methods and presented results in text, as is conventional when reporting qualitative research results.  Further, as per the guidelines in particular relation to qualitative research studies, we *“reported [our study] in accordance to the Consolidated criteria for reporting qualitative research (COREQ) checklist…”*  (Source of cited text: [PLOS ONE: accelerating the publication of peer-reviewed science](https://journals.plos.org/plosone/s/submission-guidelines#loc-qualitative-research)) | **No change to manuscript.** |
| **FROM REVIEWER #1:** |  |  |
| 4. Reviewer #1: Dear, thank you for your revision. I appreciated the revised draft and I hope to read the published work soon. | We thank Reviewer #1 for the time taken to review our manuscript and for the constructive feedback to date. We, too, hope to read the published work soon. | **No change to manuscript.** |
| **FROM REVIEWER #3:** |  |  |
| 5. Reviewer #3: The authors addressed most of my previous comments, and I feel the quality of their work has improved. Some of the points previously raised, however, are yet to be improved: I am under the impression that the Authors interpreted some of my previous [sic] comments as an attempt of applying a quantitative methodology on their study, particularly regarding their sampling choices: I assure that this is not the case. | We would like to remind Reviewer # 3 of his initial comments on the original manuscript – *“The methodology is not particularly strong however, due to the design being a qualitative research conducted on a sample from a single institution.”*  We feel there is no other way to interpret this comment accept on face value, that is, as a denigration and/or deprecation of qualitative research methodology. Consequently, this comment set the tone for all further comments by Reviewer #3, including the comments about sampling choices. | **No change to manuscript.** |
| 6. A) Sampling size. The topic of optimal sample size in qualitative research is a debated one. | We agree that the topic of optimal sample size in qualitative research is a debated one. Given that we agree on this point, we feel continuing the debate on this topic between Reviewer #3 and our research team is futile at this point. We recommend that we ‘agree to disagree’ on the approach to sample size and sampling in qualitative research. | We have made changes in regards to sample size throughout the manuscript in response to specific comments as outlined below. |
| 7. As the authors wrote in their response, it is true that qualitative research is not seeking statistical significance, and that sample size has no major impact in the quality of the evidence provided. However, while it is true that even a single observation may lead to some good quality evidence, the claim that size has at least some significance in this type of research can be seen as well grounded, to the point that other authors even proposed suggestion regarding this precise matter.   Cfr. [Malterud et al 2016] and [2: Vasileiou et al 2018]. | We concede that more information on choices made regarding sample size would benefit the reader and aid in transparency. | We have made changes in regards to sample size throughout the manuscript in response to specific comments as outlined below. |
| 8. The information on the sheer number of adherents to a qualitative research is an information a future reader may find useful to know, in the perspective of a future evaluation of the strength of the provided evidence regardless of its quality, and also, it furthers the goal of sustaining the most transparent behavioural practices in research. | While the intention of our manuscript is not to produce a methodological paper, we agree with this insightful comment and acknowledge that any explanations or rationale for choices made in research would be of benefit to the reader. | We have added further explanations on qualitative research processes used in the study to make this transparent and to benefit the reader.  ***Topic: Reflexivity***  Subheading: Data analysis  Page: 13  Lines 239-243  ***Topic: Triangulation of data from different sources***  Subheading: Discussion  Page: 33  Lines 663-665, 668-669  Subheading: Discussion  Page: 35  Lines 714-718 |
| 9. Moreover, it is hard to conceive any legitimate reason not to disclose such an information, that albeit not vital for the research, is obviously known, available and easily for the Authors to provide. Actually, the authors claim in their response that "The unit of measure is the focus group, not the individual staff member". Also in the paper, page 13 lines 241-242, they write: "Two focus groups were conducted back-to-back with a total of six and nine participants, respectively". Since such information are already provided in-text, please amend the "Results" section in the abstract accordingly. | We have amended the results section of the abstract as suggested.  In order to keep to the abstract length requirement of 300 words, we have re-crafted the entire abstract to accommodate the additional text. Abstract word count is 286. | **Changes to manuscript**  Location in manuscript:  Subheading: Abstract  Pages: 3-4  Lines: 25-47  See in manuscript. |
| 10. Also the same reasoning was behind the request of providing the information of the proportion of the size relatively to the number of eligible participants. Again, the goal has never been the one of reaching a "statistical significance", but of providing the reader with the most comprehensive information of the research itself - even more so, since focus group composition and representativeness impact in the work generalizability - a term that, for our purposes, includes both credibility and transferability of the results.   Cfr. [3: Parker and Tritter 2006] and [4: Moon et al 2016]. | We agree that providing the reader with the most comprehensive information of the research itself would enhance the manuscript. We have, thus, added more detail about our methods, findings and limitations to aid transparency and transferability of our methods. | **Changes to manuscript**  *In the following section we have provided more detail on the justification for our methodological choices regarding sample size.*  Location in Manuscript:  Subheading: Findings  Pages 13-14  Lines: 257-264  Location in Manuscript:  Subheading: Limitations  Pages: 38-39  Lines: 786-809  See in manuscript. |
| 11. B) Sampling strategy. The authors did not provide any justification in choosing a purpositive [sic] sampling strategy, simply claiming that Purposive sampling is widely used and random sampling provide no actual benefits. | We are satisfied with our previous responses to Reviewer #3 on the topic of sampling and sample size in qualitative research. We agree that adding the information in the text would enhance the manuscript. We have, thus, incorporated our previous responses into the manuscript to enhance transparency and transferability of our methods. | **Changes to manuscript**  Subheading: Participants  Page: 10  Lines: 173-183  See in manuscript. |
| 12. Such propositions are well documented and adding this information in the text would raise its overall quality. |  |  |
| 13. Still, there are other sampling strategies the authors fail to address in their reasoning of why purpositive sampling was optimal to the research goal, most compared to other nonprobability sampling strategies.   Cfr. (5: DeCarlo 2018). |  |  |
| 14. All other comments have been adequately addressed by the Authors | We thank Reviewer #3 for the time taken to review our original manuscript and subsequent revisions. | **No change to manuscript.** |
